# Changes in Visual Acuity and Dry Eye Parameters Following One Month of Scleral Lens Wear in Patients with Keratoconus: A Prospective Observational Study from Saudi Arabia

**DOI:** 10.3390/healthcare14193296

**Published:** 2026-10-03

**Authors:** Saleh R. Alshammeri

**Affiliations:** Department of Optometry, College of Applied Medical Sciences, Qassim University, Buraydah 52571, Saudi Arabia; srshmry@qu.edu.sa

**Keywords:** contact lenses, dry eye, keratoconus, Saudi Arabia, scleral lenses, visual acuity

## Abstract

Background/Objectives: To evaluate the immediate visual correction provided by scleral lens insertion and short-term changes in ocular surface parameters associated with one month of scleral lens wear in patients with keratoconus in Saudi Arabia. Methods: This prospective observational study included 18 participants with keratoconus (18 study eyes). Scleral lenses were fitted bilaterally, while one eye per participant was selected for ocular analysis; when both eyes were eligible, the eye with the higher Kmax was selected. Participants were instructed to wear ONEFIT MED^®^ scleral lenses for at least 8 h per day for one month. Outcomes included logMAR visual acuity, Ocular Surface Disease Index (OSDI), Schirmer I test, and invasive tear break-up time (ITBUT). Paired-samples t-tests were used for paired comparisons. Results: All 18 participants completed follow-up. Mean logMAR visual acuity improved immediately after lens insertion from 0.723 ± 0.550 to 0.089 ± 0.126 at baseline and from 0.760 ± 0.544 to 0.099 ± 0.126 at one month (both *p* < 0.001). Mean OSDI decreased from 45.45 ± 20.31 to 24.84 ± 11.24 (*p* < 0.001). Schirmer I increased from 14.50 ± 7.23 to 16.89 ± 7.15 mm (*p* = 0.001), and ITBUT increased from 4.94 ± 2.04 to 5.97 ± 1.74 s (*p* = 0.010). No adverse events or complications were reported. Conclusions: Scleral lenses provided substantial immediate visual correction, while one month of regular wear was associated with reduced dry eye symptoms and modest changes in tear secretion and tear-film stability. Because this was an uncontrolled observational study, the ocular-surface findings should be interpreted as associations rather than causal effects.

## 1. Introduction

Keratoconus (KC) is a progressive, bilateral, and asymmetric corneal ectatic disorder characterized by stromal thinning and corneal protrusion, resulting in irregular astigmatism, higher-order aberrations, and progressive visual impairment. The disease typically develops during adolescence or early adulthood and can substantially impair visual function and vision-related quality of life [1,2,3].

Management of keratoconus depends on disease severity and the patient’s visual needs. In the early stages, spectacles or soft contact lenses may provide adequate visual correction. However, progressive corneal irregularity often necessitates specialty contact lenses to achieve satisfactory visual rehabilitation, while corneal collagen cross-linking is primarily used to slow disease progression rather than restore vision [2,4]. Consequently, specialty contact lenses remain an essential component of keratoconus management.

Among available specialty lens designs, scleral lenses have become an increasingly important option for managing irregular corneas. These large-diameter rigid gas-permeable lenses vault the cornea and rest on the sclera, creating a fluid reservoir between the posterior lens surface and the cornea. This reservoir neutralizes anterior corneal irregularities, reduces optical aberrations, and provides a stable refractive surface, thereby improving visual performance in patients with keratoconus [5,6,7].

In addition to their optical benefits, scleral lenses may improve ocular comfort by maintaining continuous hydration of the corneal surface and enhancing tear film stability. Previous studies have reported improvements in patient-reported symptoms, visual function, and quality of life following scleral lens wear [8,9,10,11,12,13]. However, much of the available evidence originates from North America and Europe, whereas prospective data evaluating ocular surface outcomes in Middle Eastern populations remain limited. Considering the relatively high prevalence of keratoconus in Saudi Arabia, additional regional evidence is needed to better characterize the clinical changes associated with scleral lens wear in this population.

Our group previously demonstrated that Saudi patients with keratoconus exhibit impaired tear-film stability, reduced tear volume, increased dry eye symptoms, and greater meibomian gland dysfunction compared with healthy controls [14]. Specialty contact lenses, including corneal RGP, hybrid, and scleral lenses, represent important options for visual rehabilitation in keratoconus [15]. Scleral lenses, in particular, have demonstrated improvements in visual acuity and patient-reported outcomes, supporting their role in the management of keratoconus [16]. Contact lens fitting may also influence tear-film dynamics and ocular-surface interactions [17]. Building on those findings, the present prospective study evaluates the immediate optical benefit of scleral lens insertion and short-term changes in both patient-reported symptoms and objective ocular-surface measures after one month of regular bilateral scleral lens wear. The study also provides clinical context for comparison with other contact-lens modalities used in keratoconus, including corneal rigid gas-permeable (RGP) and hybrid lenses [18,19,20].

## 2. Materials and Methods

This prospective observational study was conducted at the Optometry Clinics, Department of Optometry, College of Applied Medical Sciences, Qassim University, Saudi Arabia. Consecutive patients with clinically diagnosed keratoconus were recruited and followed for one month after scleral lens fitting. The study adhered to the tenets of the Declaration of Helsinki and was approved by the Institutional Review Board of Qassim University (Approval No. 14-25-12; 20 February 2025). Written informed consent was obtained from all participants before enrollment.

A total of 18 participants (18 study eyes) aged 19–36 years were included, comprising 10 males (55.6%) and 8 females (44.4%). The mean age was 25.4 ± 4.9 years. The study population generally represented moderate-to-advanced keratoconus, with a mean Kmax of 55.53 ± 6.22 D, mean thinnest corneal thickness of 409.05 ± 60.35 μm, and mean spherical equivalent refractive error of −9.01 ± 2.56 D. At baseline, 55% of participants primarily used spectacles for visual correction and the remaining 45% used RGP contact lenses. RGP users discontinued lens wear for one week before baseline assessment. Two participants (11.1%) had undergone corneal cross-linking more than one year before enrollment; no participant had undergone other keratoconus-related surgical procedures, including intracorneal ring segment implantation. One study eye per participant was analyzed to avoid inter-eye correlation. When both eyes met the eligibility criteria, the eye with the higher Kmax value was selected as the study eye.

All participants were fitted bilaterally with ONEFIT MED^®^ scleral lenses (Blanchard Contact Lenses, Manchester, NH, USA) according to the manufacturer’s fitting guidelines. Lens parameters, including diameter and sagittal depth, were individualized according to the clinical fitting assessment rather than fixed across participants. Central corneal clearance was assessed clinically to ensure an acceptable fit; quantitative clearance values were not recorded. Final lens power was individualized during the fitting process but was not systematically collected as a study variable. Final fit acceptance was based on adequate central and limbal clearance and an appropriate scleral landing without clinically significant compression or impingement. Participants were instructed to wear the lenses for at least 8 h per day during the one-month follow-up. Compliance with the recommended wearing schedule was assessed by direct patient questioning at the follow-up visit.

Subjective dry eye symptoms were assessed using the Ocular Surface Disease Index (OSDI) at the participant level, reflecting symptoms during bilateral scleral lens wear. Tear-film stability was assessed using invasive tear break-up time (ITBUT), and tear secretion was evaluated using the Schirmer I test without topical anesthesia, with the wetted length recorded after five minutes. Baseline and one-month follow-up assessments were scheduled between 10:00 a.m. and 12:00 p.m. At the one-month visit, scleral lenses were removed and a 10 min interval was allowed before ocular-surface assessment. Testing was performed in a standardized sequence from the least to the most invasive procedure: OSDI first, followed by ITBUT, and finally the Schirmer I test. Visual acuity, Schirmer I, and ITBUT were analyzed at the study-eye level, whereas OSDI was analyzed at the participant level.

The primary visual outcome was the immediate change in visual acuity following scleral lens insertion, assessed at the baseline fitting visit and again at the one-month follow-up visit. Longitudinal secondary outcomes were changes in OSDI score, Schirmer I test values, and ITBUT from baseline to one month. An a priori sample size calculation was performed using G*Power software version 3.1.9.7 (Heinrich Heine University Düsseldorf, Düsseldorf, Germany) for the paired pre–post study design. Assuming a large effect size (Cohen’s d = 0.75), a two-sided α of 0.05, and 80% statistical power, the minimum required sample size was 16 participants; the final sample of 18 participants exceeded this requirement. Statistical analyses were performed using IBM SPSS Statistics version 26.0 (IBM Corp., Armonk, NY, USA). Data normality was assessed using the Shapiro–Wilk test and was considered appropriate for parametric paired analysis. Continuous variables are presented as mean ± standard deviation (SD). Paired-samples *t*-tests were used for paired comparisons. All tests were two-sided, and *p* < 0.05 was considered statistically significant.

## 3. Results

All 18 participants completed the one-month follow-up, with no loss to follow-up. The sample comprised 10 males (55.6%) and 8 females (44.4%), with a mean age of 25.4 ± 4.9 years. Mean Kmax was 55.53 ± 6.22 D, mean thinnest corneal thickness was 409.05 ± 60.35 μm, and mean spherical equivalent was −9.01 ± 2.56 D. At baseline, 55% primarily used spectacles and 45% used RGP contact lenses for visual correction. Two participants (11.1%) had undergone CXL more than one year before enrollment. No other keratoconus-related surgical procedures were reported.

**Figure 1:**
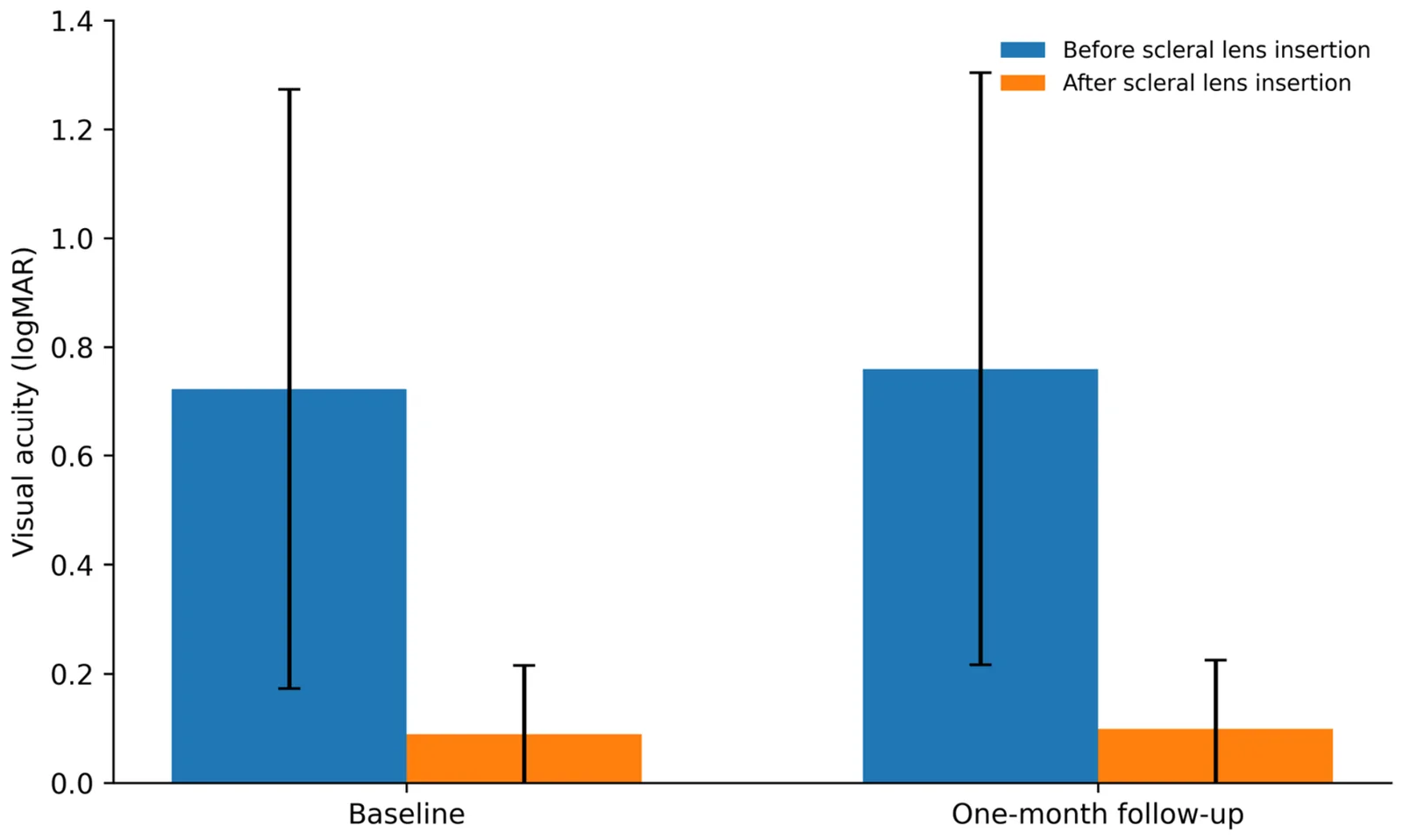
Mean logMAR visual acuity before and immediately after scleral lens insertion at the baseline fitting visit and the one-month follow-up visit. Error bars represent standard deviations. Lower logMAR values indicate better visual acuity. At both visits, visual acuity improved significantly immediately after lens insertion (*p* < 0.001).

Visual acuity, expressed as logMAR, demonstrated a marked immediate improvement after scleral lens insertion at both visits. At baseline, mean visual acuity improved from 0.723 ± 0.550 before lens insertion to 0.089 ± 0.126 immediately after insertion (*p* < 0.001). At the one-month visit, mean visual acuity improved from 0.760 ± 0.544 before lens insertion to 0.099 ± 0.126 immediately after insertion (*p* < 0.001). These comparisons represent the immediate optical benefit of scleral lens correction at each visit rather than a cumulative one-month improvement in unaided visual acuity. The visual acuity results are summarized in Table 1 and illustrated in Figure 1.

Subjective dry eye symptoms decreased after one month of scleral lens wear. Mean OSDI score decreased from 45.45 ± 20.31 at baseline to 24.84 ± 11.24 at one month (*p* < 0.001). The post-wear mean remained within the moderate symptom range. Because OSDI is a participant-level measure, this change reflects symptoms during bilateral scleral lens wear.

Mean Schirmer I values increased from 14.50 ± 7.23 mm at baseline to 16.89 ± 7.15 mm after one month (*p* = 0.001). Although statistically significant, the absolute change was modest and should not be interpreted as evidence of a causal enhancement of tear production in the absence of a control group.

Mean ITBUT increased from 4.94 ± 2.04 s to 5.97 ± 1.74 s after one month (*p* = 0.010). This statistically significant change was modest, and the post-wear mean remained below the conventional normal range. Detailed ocular-surface outcomes are summarized in Table 2.

No adverse events or complications related to scleral lens wear were reported during the one-month follow-up period.

## 4. Discussion

This prospective observational study evaluated the immediate optical benefit of scleral lens insertion and short-term ocular-surface changes associated with one month of regular bilateral scleral lens wear in patients with keratoconus. Scleral lens insertion produced substantial immediate improvement in visual acuity at both visits, while one month of wear was associated with lower OSDI scores and modest increases in Schirmer I and ITBUT. All participants completed follow-up, and no adverse events or complications were reported during the study period.

The immediate visual improvement is consistent with the established ability of scleral lenses to mask irregular corneal astigmatism and provide a stable refractive surface [5,9,12,15]. Importantly, the pre- versus post-insertion comparisons quantify the immediate optical benefit at each visit and should not be interpreted as evidence of a cumulative one-month improvement in unaided visual acuity. Hybrid contact lenses have likewise been reported to improve visual acuity in advanced keratoconus, illustrating that substantial optical rehabilitation can be achieved with different specialty lens designs [20].

Participants also reported lower dry eye symptom scores after one month. This observation is consistent with reports of improved ocular comfort with scleral lenses [10,16]. In contrast, RGP lens studies in keratoconus have described less favorable ocular-surface findings. Yüksel Elgin et al. reported differences in OSDI, Schirmer, and tear break-up measures among RGP wearers, non-wearers, and healthy controls [18], while Carracedo et al. found greater dry-eye symptoms and lower tear volume and stability among RGP-wearing keratoconus patients than non-wearers [19]. These studies are not directly equivalent to the present prospective scleral-lens design, but they provide useful context suggesting that ocular-surface responses may vary with lens modality and fitting relationship.

Schirmer I and ITBUT increased after one month, but the absolute changes were modest, and the ITBUT mean remained below the conventional normal range. Accordingly, these findings should be interpreted as short-term changes associated with scleral lens wear rather than evidence that scleral lenses directly increase tear production or normalize tear-film function. The absence of a control group is particularly important when interpreting these outcomes, because temporal variation, adaptation, and other unmeasured factors cannot be excluded.

Although the present study used the ONEFIT MED design, some of the observed symptomatic benefits may plausibly extend to other modern scleral lens designs because corneal vaulting and a fluid-filled post-lens reservoir are shared principles of scleral lens wear. Carracedo et al. reported a significant reduction in OSDI after 8 h of scleral lens wear in keratoconus, although Schirmer and tear break-up time did not change significantly [21]. Broader reviews of scleral lens effects on the tear film and dry eye similarly support symptomatic relief and ocular-surface protection, while emphasizing that the evidence remains heterogeneous [22,23]. More recently, a randomized pilot study comparing mini-scleral and corneal RGP lenses in patients with irregular corneas and dry eye found improvement in dry-eye symptom scores with both modalities, with mini-scleral lenses showing favorable subjective comfort, while TBUT did not differ significantly [24]. Therefore, similar symptom improvement may be expected with other appropriately fitted scleral or mini-scleral lenses, but the magnitude of objective tear-film changes should not be assumed to be identical across designs. Lens diameter, sagittal depth and post-lens reservoir thickness, haptic/landing-zone geometry, material oxygen permeability, fenestration or ventilation, tear exchange, surface wettability, and fitting characteristics can modify ocular-surface physiology and patient experience [7,22,23]. Accordingly, the present findings should be generalized to other scleral lens designs cautiously, and direct comparative studies are needed.

The present study extends our previous cross-sectional work describing ocular-surface abnormalities in Saudi patients with keratoconus [14] by adding prospective short-term observations after scleral lens fitting. Its contribution therefore lies not simply in geographic novelty, but in the combined assessment of immediate visual correction, participant-reported symptoms, and objective tear parameters over one month of bilateral scleral lens wear in a clinically characterized keratoconus cohort.

Strengths of this study include its prospective design, use of both patient-reported and objective outcomes, standardized examination timing and testing sequence, and examination by the same experienced examiner. Limitations include the small sample size, absence of a control group, and one-month follow-up, which limit precision, generalizability, and causal inference. In addition, quantitative scleral lens fitting parameters such as sagittal depth, central clearance, and final lens power were not systematically collected as study variables, and compliance was assessed by direct patient questioning rather than an objective wear-time measure. Individual keratoconus staging according to Amsler–Krumeich or Belin ABCD was not prospectively recorded; therefore, severity was characterized using the available Kmax and thinnest corneal thickness data rather than retrospectively assigning stages.

## 5. Conclusions

This prospective observational study showed that scleral lenses provided marked immediate visual improvement in patients with keratoconus and that one month of regular bilateral wear was associated with a substantial reduction in patient-reported dry eye symptoms and modest increases in Schirmer I and invasive tear break-up time. Because the study was small, uncontrolled, and limited to one month of follow-up, the ocular-surface changes should be interpreted as short-term associations rather than causal effects. Larger controlled studies with longer follow-up and more comprehensive ocular-surface assessment are warranted to determine the durability and clinical significance of these findings and their applicability across different scleral lens designs.

## Figures and Tables

**Table 1:** Values are presented as mean ± standard deviation (SD). Lower logMAR values indicate better visual acuity. *p* values were calculated using paired-samples *t*-tests.

Visit	Before Scleral Lens Insertion (Mean ± SD)	After Scleral Lens Insertion (Mean ± SD)	*p* Value
Baseline	0.723 ± 0.550	0.089 ± 0.126	*p* < 0.001
One-month follow-up	0.760 ± 0.544	0.099 ± 0.126	*p* < 0.001

**Table 2:** OSDI = Ocular Surface Disease Index; ITBUT = invasive tear break-up time. Values are presented as mean ± standard deviation (SD). *p* values were calculated using paired-samples *t*-tests.

Outcome	Baseline (Mean ± SD)	One-Month Follow-Up (Mean ± SD)	*p* Value
OSDI score	45.45 ± 20.31	24.84 ± 11.24	*p* < 0.001
Schirmer I (mm)	14.50 ± 7.23	16.89 ± 7.15	*p* = 0.001
ITBUT (s)	4.94 ± 2.04	5.97 ± 1.74	*p* = 0.010

## Data Availability

The data presented in this study are available on request from the corresponding author. The data are not publicly available due to participant privacy and ethical restrictions.
